# Ocins database: a database of bug-busters from *Bifidobacterium*, *Lactobacillus*, and *Enterococcus*

**DOI:** 10.1099/acmi.0.000034

**Published:** 2019-06-13

**Authors:** Shilja Choyam, Suresh PSN, Rahul Pandey, Rajagopal Kammara

**Affiliations:** ^1^ Department of Protein Chemistry and Technology, CSIR-CFTRI, Mysore, Karnataka 570020, India; ^2^ AcSIR, Department of PCT, CSIR-CFTRI, Mysore, Karanataka, India; ^3^ Computer Centre Facility, CSIR-CFTRI, Mysore, Karanataka, India; ^4^ Food Technology Dissertation student, Mysore, Karanataka, India

**Keywords:** Ocin, bacteriocin, enterocin, lactocin, lactic acid bacteria, Bifidobacteria

## Abstract

The ocins are antimicrobial polypeptides produced by probiotic microbes, such as *Lactobacillus *, *Enterococcus *, *Streptococcus *, *Leuconostoc* and *Bifidobacterium *. They are produced in response to stress and for the self-defense of the bacterium. It is indispensable to understand their mechanistic characteristics, structures, and functions, if the food industry is to reduce contamination levels and produce germfree foods. Databases of the ocins that are readily accessible to the food industry are scarce, but urgently required. Therefore, we established a very useful, unique, and a simple ocin database, which not merely provides information about ocins, but also directs their utilisation in the food industry. The database includes information about each ocin, its amino acid sequence, molecular weight, and isoelectric point. The database also possesses all the currently known ocin (probiotic origin only) sequences and structures, target organisms, and relevant to food industries (aqua culture, dairy and meat industries), which is hard to obtain in other databases. The database is free for public and accessed at http://ocins.cftri.com/ocins/.

## Introduction

In the late 19th century, microbiologists identified differences in the microbial contents of the gastrointestinal tracts of healthy and diseased individuals. The beneficial microorganisms found in the gastrointestinal tract were designated ‘probiotics’, literally meaning ‘for life’. Probiotics are living microorganisms that beneficially influence the health of humans when consumed in adequate numbers (10^9^ to 10^11^) [1]. The Nobel laureate Metchnikoff is credited with recognising the health benefits of probiotics in 1908 [2], suggesting that the consumption of living lactic acid bacteria in fermented foods improves health and longevity by favorably modulating the GI (gastrointestinal) microflora. Over the past century, an increasing body of basic and clinical research has supported the health benefits of probiotic consumption in a host of disorders, including irritable bowel syndrome, inflammatory bowel disease, diarrhea, food allergies, lactose intolerance, urogenital infections, and atopic eczema [3–6]. *Bifidobacterium* and *Lactobacillus* are the principal genera of probiotic microorganisms. Later developments in this field have shown that other types of bacilli and yeasts (*Saccharomyces cerevisiae, Saccharomyces boulardii*) can also be considered probiotics. For a bacterial strain to be considered a probiotic, it must satisfy the following conditions: ability to grow at a low pH, ability to grow in the presence of bile salts, antimicrobial function, non-toxicity and non-pathogenic in nature [7].

Current literature suggests that probiotics enhance both the specific and nonspecific immune responses, by activating macrophages or increasing natural killer cell activity, or increasing cytokine and/or immunoglobulin levels [8–10]. Most probiotic organisms are commensals in nature (living in the human gut) and are commonly consumed in fermented foods, such as yogurt, cheese, kefir, sauerkraut, etc. The established use of probiotics for their health benefits becomes more striking because these bacteria produce bacteriocins [11], proteinacious toxins that inhibit the growth of similar bacterial strains [12]. *In vivo* studies have shown that bacteriocin production improves the successful establishment of the producing strains. These molecules display significant antimicrobial activity against other bacteria (including antibiotic-resistant strains), are stable, and have narrow- or broad-spectrum activity. Bacteriocins can even be produced *in situ* in the gut by probiotic bacteria to combat intestinal infections. Recent research in bacteriocins has identified various bacteriocins that can be used as food preservatives or as bactericidal agents against pathogenic microorganisms [13–15]. Nisin is one of the most-studied bacteriocins in the field of food preservation [16]. These bacteriocins are nontoxic to humans and are widely used in food preservation. They have been identified based on their origin. Enterocin is produced by *Enterococcus* spp., lactocin by *Lactococcus* spp., and bacteriocin by *Bifidobacterium* spp. Therefore, these three types of proteins are collectively called the ‘ocins’. Because of their wide range of applications in the food industry, it is important to arrange ocins systematically in terms of their intended use. As per their mechanism and action all the ocins have the ability to kill the pathogens. Therefore, they may also refer to ‘bug-busters’. This has guided us to produce a novel bacteriocin database for the specific support of food manufacturers as well as researchers. However, other databases also list important ocins, including BACTIBASE and BAGEL [17–19]. But, they do not show any relevance to the food industry.

In the BACTIBASE system, ocins are grouped according to their structure and mode of action. The ocins are arranged in a coherent manner based on the phylogenetic relationships between different ocins. Understanding the phylogenetic relationships of ocins and how they correlate with their structures allows the BACTIBASE data to be arranged according to their evolutionary status. However, only 70 % of the data can be assembled. The BACTIBASE database provides an overview of 177 ocins [20]. Since the establishment of BACTIBASE, very large numbers of ocins have been discovered that are not already incorporated into the existing databases. Furthermore, BACTIBASE is based on the morphological similarities between ocins. It is difficult to identify antimicrobial ocins in the database that are specifically useful in the prevention of food contamination by organisms such as *Salmonella *, *Vibrio cholerae *, and *Listeria monocytogenes *. DBAASP (http://dbaasp.org) [21] is the recently developed antimicrobial database. It is the database of antimicrobials that predicts activity and their structure. DRUMP (http://dramp.epu-bioinfor.org) [22] is a structure prediction software, and information portal to biologically active peptides. CAMP (http://www.camp3.bicnirrh.res.in/) [23] refers to the collection of antimicrobial peptides, the major objective of the database is to discover and design novel antimicrobial peptides. LAMP is a database made for linking antimicrobial peptides. The major objective of the database is (http//biotechlab.fudan.edu.cn/database/lamp) [24] discovery and design of antimicrobials as new antimicrobial agents. Although various antimicrobial polypeptide databases exist, none of them are specifically made in the food industry specifying the antimicrobial agents their source, and their target, etc. Therefore, ocins database has been created and dedicated to the food industry (http://ocins.cftri.com/ocins/).

There has been no report of an antimicrobial database that describes the actions of specific antimicrobial ocins against food-contaminating pathogens. The major objective of the database is the direct application of antimicrobial agents in food manufacture. In the ocins database, food groups were arranged in the form of a pyramid. We established the food pyramid to represent a healthy diet for any individual globally. The foods are divided into the following categories such as food grains and cereals, fruits and vegetables, meat and meat products derived from pig, buffalo, sheep, goats, birds, fish and fish products, milk and milk products (dairy) and fats. We then sub-categorised each food group, according to the different types of pathogenic bacteria that can possibly infect. These spoilage and pathogenic bacteria can be controlled by a number of ocins. Therefore, at this point, the database moves directly to the ocins that are active against specific pathogenic organisms. Thus, the user gains a broad picture of the different kinds of food groups, the pathogenic organisms that infect them, and the ocins that inhibit/control these organisms. The inclusion of these kinds of data makes the database quite unique.

## Methods

The ocin peptide sequences were obtained from different sources: the UniProt database, PubMed (from the relevant publications), and BACTIBASE. Because not all known ocin sequences are available in the ExPASY (ExPASy SRS server [http://www.expasy.org/srs/]), the National Center for Biotechnology Information (NCBI) server (http://www.ncbi.nlm.nih.gov/entrez/), BACTIBASE, or ANTIMIC databases. A selection of them that have been isolated from recent reports ‘and validated in our own laboratory’ have been included. A literature search was used to complete the ocins database, which only contains natural mature peptides, and does not contain a signal or leader sequences. Each sequence entry contains general data, including the peptide name, sequence, class, etc. The physico-chemical data presented for each peptide includes the mass, length, pI, net charge, hydropathy profile, amino acid profile, thermostability, thermolability, resistance or susceptibility to various physiological enzymes, cysteine contents, and wheel diagram.

Information pertaining to ocins was mined from databases such as NCBI, EMBL, DDBJ, and BACTIBASE. The BACTIBASE database was thoroughly perused to extract the information relevant to ocins. Exhaustive literature searches were made of the PubMed database using keywords such as ‘antimicrobials from probiotics’, ‘bacteriocin, lactocin and enterocin’, and ‘antifungal and antibacterial polypeptides/peptides’, which produced 524 results. The sequences available were examined and sorted based on validated reports, which narrowed the entries to 222 sequences containing potentially validated data. These articles were then validated in detail manually. Precedence was given to articles with experimentally validated sequences. Review articles were considered, however non-English articles were not considered.

The Java platform was used to visualize 3D protein structures (predicted) and wheel structures. The desired ocin sequences were collected from each peer-reviewed and published article (and from BACTIBASE). The data were arranged in rows and columns using a Microsoft Excel file. Each column contains data representing the sequence and characteristics of the ocin.

The following themes define the database schema:

1. UniProt ID: Unique identification number was included for each record in the UniProt database.
2. Sequence: All protein sequences are formatted with the standard single-letter amino acid notation. Amino acid sequences are displayed in the FASTA format.
3. Nomenclature: The original name as it is cited in the literature.
4. References: For each sequence, a reference to the abstract of the corresponding article was given.

**Fig. 1.**
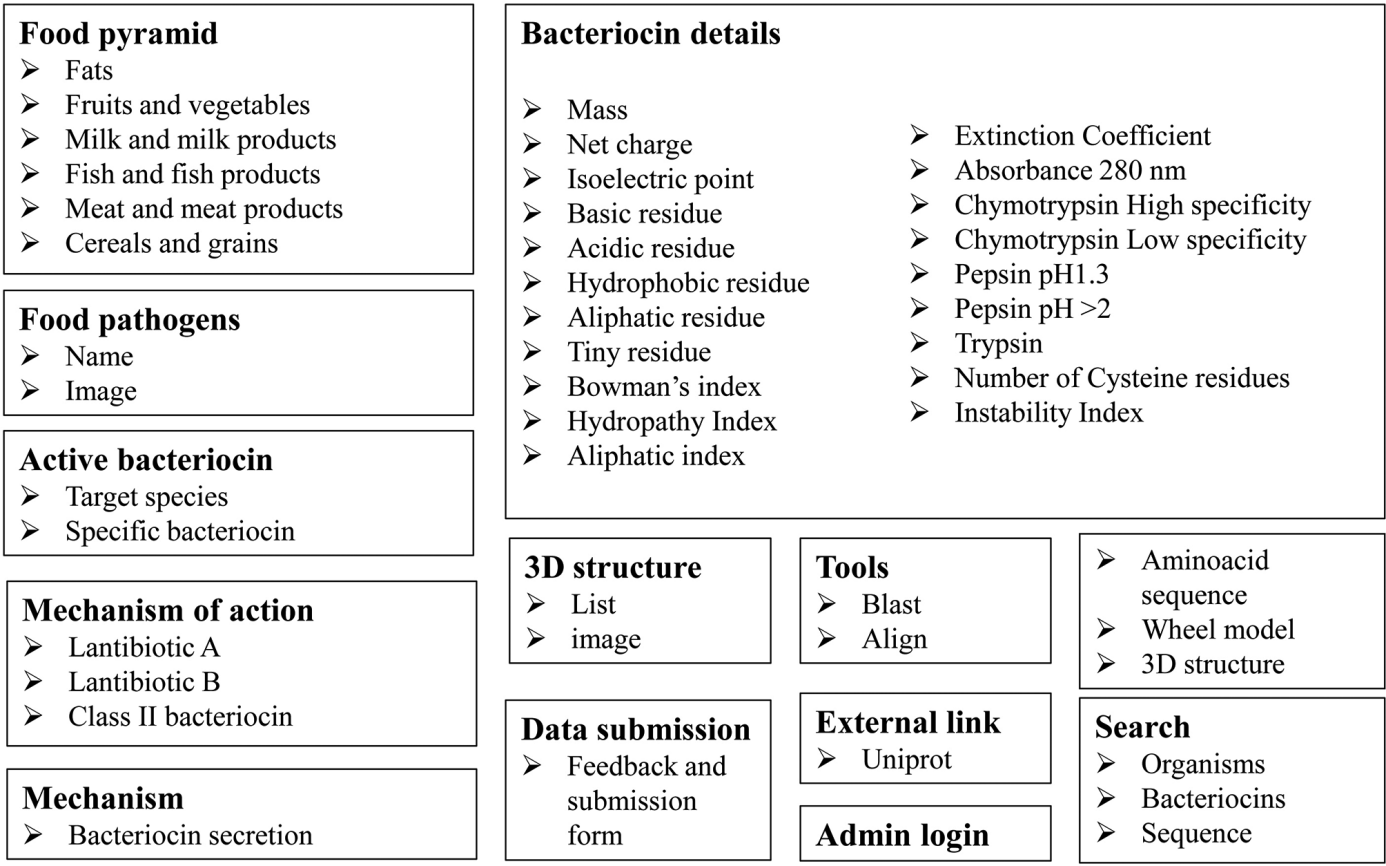
The basic components of Ocins Database.

**Fig. 2.**
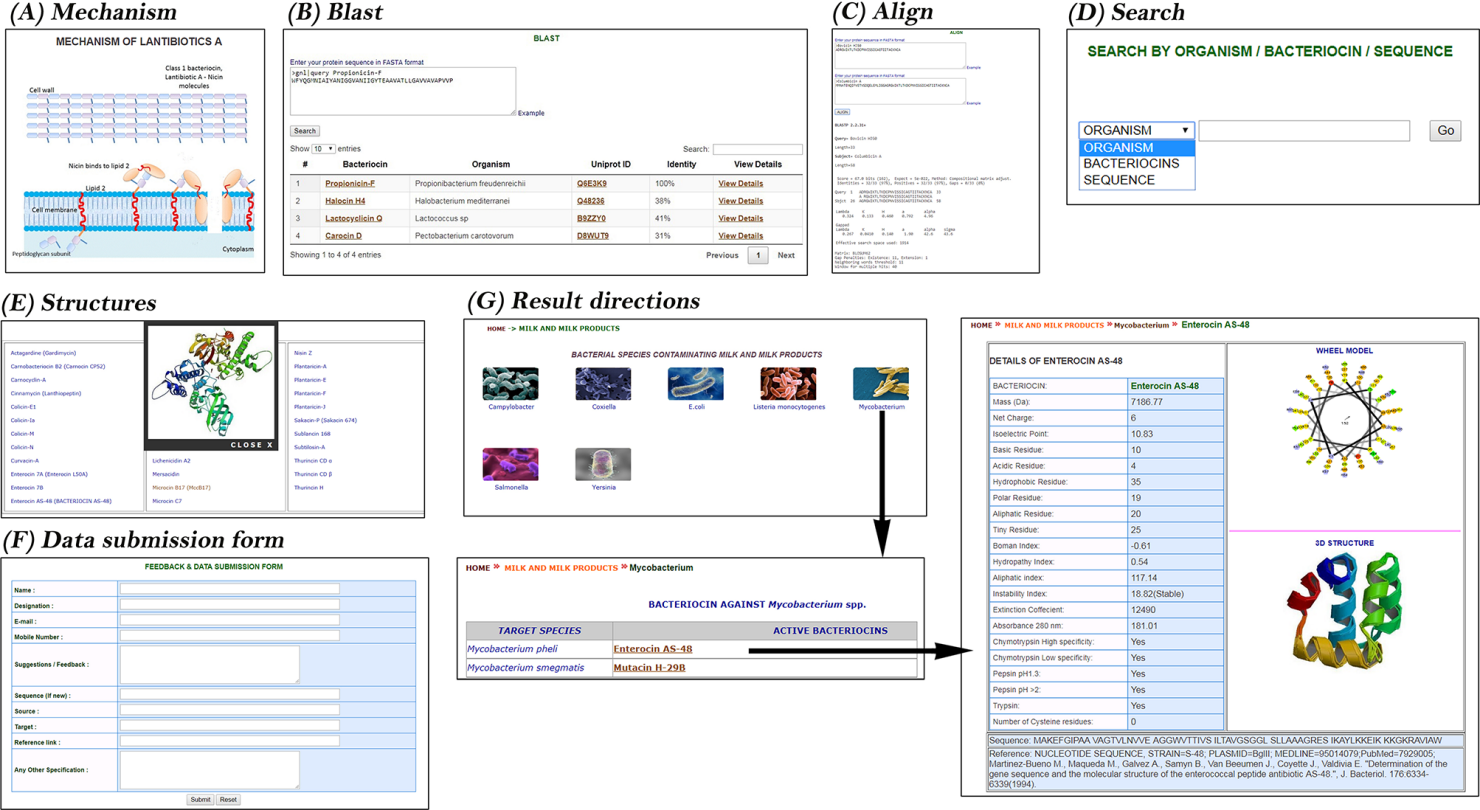
The schematic work flow of Ocins database. The options provided on the home page of the database includes mechanism (a), which provides mechanism of bacteriocin action its secretion, Blast (b), align (c), browse (d), structures (e), data submission form (f). The results directions (g) shows the search links directly from the food categories to the spoilage microbes and to bacteriocin details.

In summary, the other characteristics, such as the structural and physicochemical properties, was accessed at the corresponding locations. No particular effort has been made to analyse the hydrophobicity of the ocins, and very few ocin (predicted) structures have been reported and deposited in the protein (PDB) structural database. However, all the available and reported structures (predicted) had been integrated in the ocins database. The basic components of ocin database were shown in Fig. 1, which includes all the information gathered and its arrangements in various titles. The schematic workflow and the utilisation of the database resource were shown in Fig. 2, including various search options (Blast, Align, structures, feedback and submission form and mechanisms) and a main link to the bacteriocins through food pyramid. The food pyramid leads to the food pathogens page and then to the bacteriocins details.

### Database architecture and web interface

The database design includes simple and dynamic user interface, detailed information inputs and retrieval, regular updates from the admin, modifications, checking for redundancy etc. The structural design of ocin database is shown in figure (Fig. S1, available in the online version of this article).

- Admin can login and insert the data in the Information Server, which will be connected to the router through LAN
- Intranet users can retrieve the data from the web page
- Based on the input, the user will get the details of the pathogenic organisms specific to the food categories. Bacteriocin specific to the target species and all details of the bacteriocins along with Reference and Sequence.

### Implementation of the ocin database

The aim of automation is to integrate the usage of ocin database, bacteriocins through an easily accessible platform. Ocin database is a DNA and protein repository (of ocins) that contain the information about their nucleic acid, amino acid sequences, and various physico-chemical parameters. The database may be very useful for the global scientific community in the field as well as for the food industries to make germfree foods.

### The implementation of ocin database consists of the following sections as shown in Figure (Fig. S1b)

- Admin
- User

Admin section will maintain and monitor the database by inserting, viewing, updating, organism and bacteriocins data etc. related to ocin database.

In User section, the user inputs / search the organism, bacteriocins name and sequence details the program automation will compare with the existing data from the database and sends the automated results.

Intel Core i7-4770 CPU 3.40 GHz (HP) systems were used to develop the database and for trial runs. The Apache 2.0 web server was used to host the website. The available data from NCBI was used and further refined with data available in the public domain. The programming languages viz., HTML, PHP, JS and MySQL were used for developing the databases. The database schema is conceptualised and rationalised, which includes admin log in, add, update, view, retrieval, display, etc. The schema of ocin database is presented in figure (Fig. S1).

### User interface (data access)

Blast, align, and search services allow the users to query the database. Three different categories can be followed in searching the database, and information pertaining to them can be retrieved using the relevant keywords: (i) organism name (ii) bacteriocin name and, (iii) bacteriocin sequence.The search page is extremely useful when looking for specific information in the whole database. The specific search page allows the use of more flexible and specific queries, with logical operators (AND, OR). This function also allows the appropriate data to be found more rapidly.

### Blast and align

The bacteriocin sequences can be blasted, and aligned against the existing sequences of the bacteriocins. The external link for the same bacteriocin is also provided here.

### Data submission form

The authors wish to submit new ocin sequences may contact the curator/administrator at rajagopalk@cftri.res.in, krgopal22@rediffmail.com. Once the sequences are analysed and validated, they will be automatically incorporated into the system by the concerned. Only the curator of the database can upload raw data after its systematic validation.

## Results and Discussion

### Highlighting the YGNGV family proteins

Pediocin is other antimicrobial peptide that is extensively used, after nisin. The basic feature of pediocin is the presence of the Y-G-N-G-V motif, and they specifically act on Listeria spp. Therefore, they are anti-Listerial peptide family. The dairy industry is highly affected because of Listerial infections/contaminations. Therefore, it is essential to understand the significance of YGNGV family. We analysed 29 proteins (obtained from different databases such as BACTIBASE, APD etc.) containing 26–74 amino acid residues that represent the YGNGV family (Table 1). A multiple sequence alignment identified 5–7 highly conserved amino acid residues. The sequences surrounding the YGNGV motif are conserved. Two cysteine residues upstream from the YGNGV motif form a disulfide bond, and are also conserved. Only one ocin contains a PGNGV region, in which proline replaces tyrosine. Regardless of any differences in the amino acid sequence, the overall charge is always maintained. The space between the two cysteine residues is constant, and contains seven amino acid residues. Thirteen ocins contain one positively charged amino acid residue between the cysteine residues, and the rest are neutral. The YGNGV family proteins are secreted in their active forms, and target the microbial cell wall.

**Table 1.** Twenty nine different ocins showing their entry name, protein name, coding gene, source and length

Entry name	Protein names	Gene names	Organism	Length
BACT_STRMU	**Bacteriocin mutacin F-59.1**		* Streptococcus mutans *	22
BCN37_PAEPO	**Bacteriocin SRCAM 37**		* Paenibacillus polymyxa ( Bacillus polymyxa *)	30
CURVA_LACCU	**Bacteriocin curvaticin**		* Lactobacillus curvatus *	30
BCN80_BACCI	**Bacteriocin SRCAM 1580**		* Bacillus circulans *	35
LCNM_LACLL	**Bacteriocin lactococcin MMFII**		* Lactococcus lactis subsp. lactis* (* Streptococcus lactis *)	37
ETCM_ENTFC	**Bacteriocin enterocin-M**		* Enterococcus faecium* ( Streptococcus faecium )	38
BCN02_PAEPO	**Bacteriocin SRCAM 602**		* Paenibacillus polymyxa* (* Bacillus polymyxa *)	39
ETC50_ENTFC	**Bacteriocin E50-52**		* Enterococcus faecium* (* Streptococcus faecium *)	39
BACT_LACSP	**Bacteriocin**		Lactococcus sp.	41
BAVA_LACSK	**Bacteriocin bavaricin-A**		* Lactobacillus sakei *	41
BAVM_LACSK	**Bacteriocin bavaricin-MN**		* Lactobacillus sakei *	42
DIV35_CARDV	**Bacteriocin divergicin M35**		* Carnobacterium divergens* (* Lactobacillus divergens *)	43
ETCHF_ENTFC	**Enterocin-HF**		* Enterococcus faecium* (* Streptococcus faecium *)	43
LCCC_LEUME	**Bacteriocin leucocin-C**		* Leuconostoc mesenteroides *	43
MUTI_ENTMU	**Bacteriocin mundticin**		* Enterococcus mundtii *	43
WSNA_WEIPA	**Bacteriocin weissellin-A**		Weissella paramesenteroides (* Leuconostoc paramesenteroides *)	43
SAKA_LACCU	**Bacteriocin curvacin-A**	**curA**	* Lactobacillus curvatus *	59
SAKA_LACSK	**Bacteriocin sakacin-A**	**sapA** sakA	* Lactobacillus sakei *	59
CBB1_CARML	**Bacteriocin carnobacteriocin BM1**	**cbnBM1**	* Carnobacterium maltaromaticum* ( Carnobacterium piscicola )	61
LCCA_LEUGE	**Bacteriocin leucocin-A**	**lcnA**	* Leuconostoc gelidum *	61
LCCB_LEUCA	**Bacteriocin leucocin-B**		* Leuconostoc carnosum *	61
MTCY_LEUME	**Bacteriocin mesentericin Y105**	**mesY**	* Leuconostoc mesenteroides *	61
SAKP_LACSK	**Bacteriocin sakacin-P**	**sakP** sakR sppA	* Lactobacillus sakei *	61
PIS1_CARML	**Bacteriocin piscicolin-126**	**pisA**	* Carnobacterium maltaromaticum* ( Carnobacterium piscicola )	62
PPA1_PEDAC	**Bacteriocin pediocin PA-1**	**pedA** pap papA	* Pediococcus acidilactici *	62
CBB2_CARML	**Bacteriocin carnobacteriocin B2**	**cbnB2** canCP52	* Carnobacterium maltaromaticum* ( Carnobacterium piscicola )	66
UBAA_STRUB	**Bacteriocin ubericin-A**	**ubaA**	* Streptococcus uberis *	70
ETCP_ENTFC	**Bacteriocin enterocin-P**	**entP**	* Enterococcus faecium* ( Streptococcus faecium )	71
HJM79_ENTHR	**Bacteriocin hiracin-JM79**	**hirJM79**	* Enterococcus hirae *	74

Various Ocins refered in the present study.

**Fig. 3.**
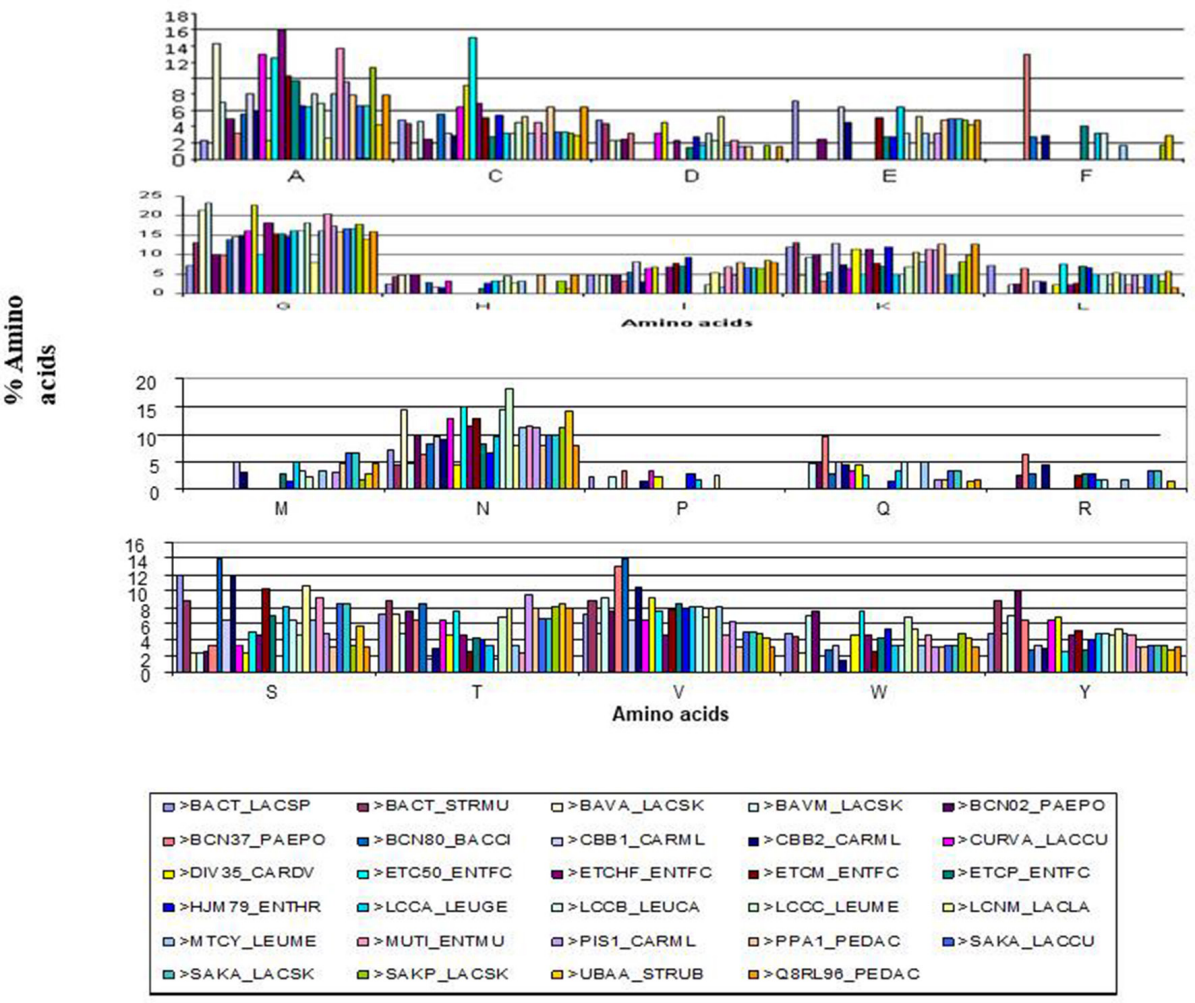
Distribution of amino acid residues. The Perl Script program was used to calculate the amino acid distribution patterns in 29 different ocins. The chromatogram shows two parameters: the X-axis corresponds to the amino acid residues and the Y-axis to their occurrence (%). The distribution of each amino acid is shown.

**Fig. 4.**
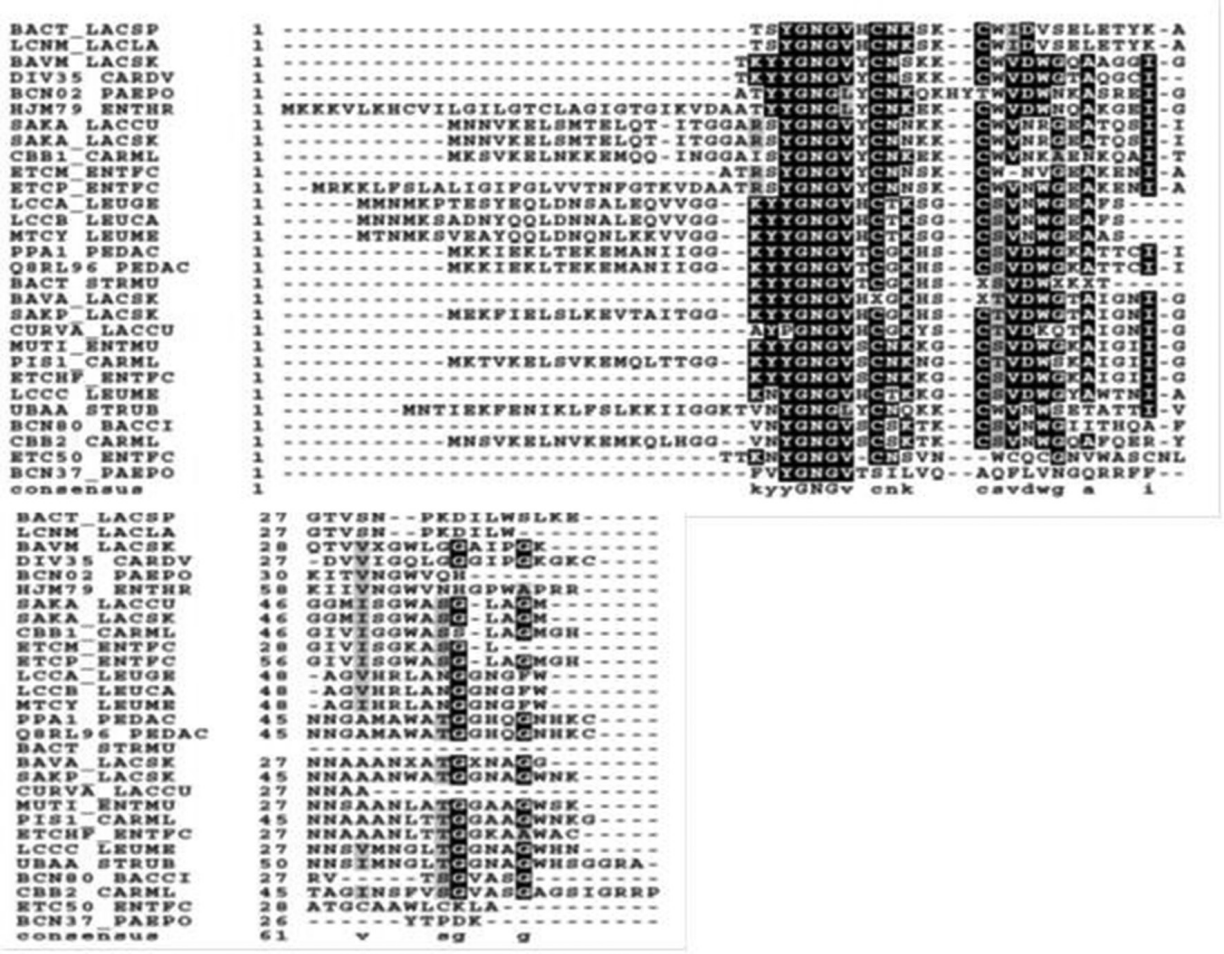
Sequence alignment to show YGNGV sequences. Amino acid sequences of the YGNGV family ocins were aligned to ClustalX version 1.83. Highly conserved amino acid residues are shaded in dark gray and the sequence is shown at the bottom. Semi conserved sequences are light, dark shaded and they are shown at the bottom. This alignment was generated with the UniProt alignment program.

**Fig. 5.**
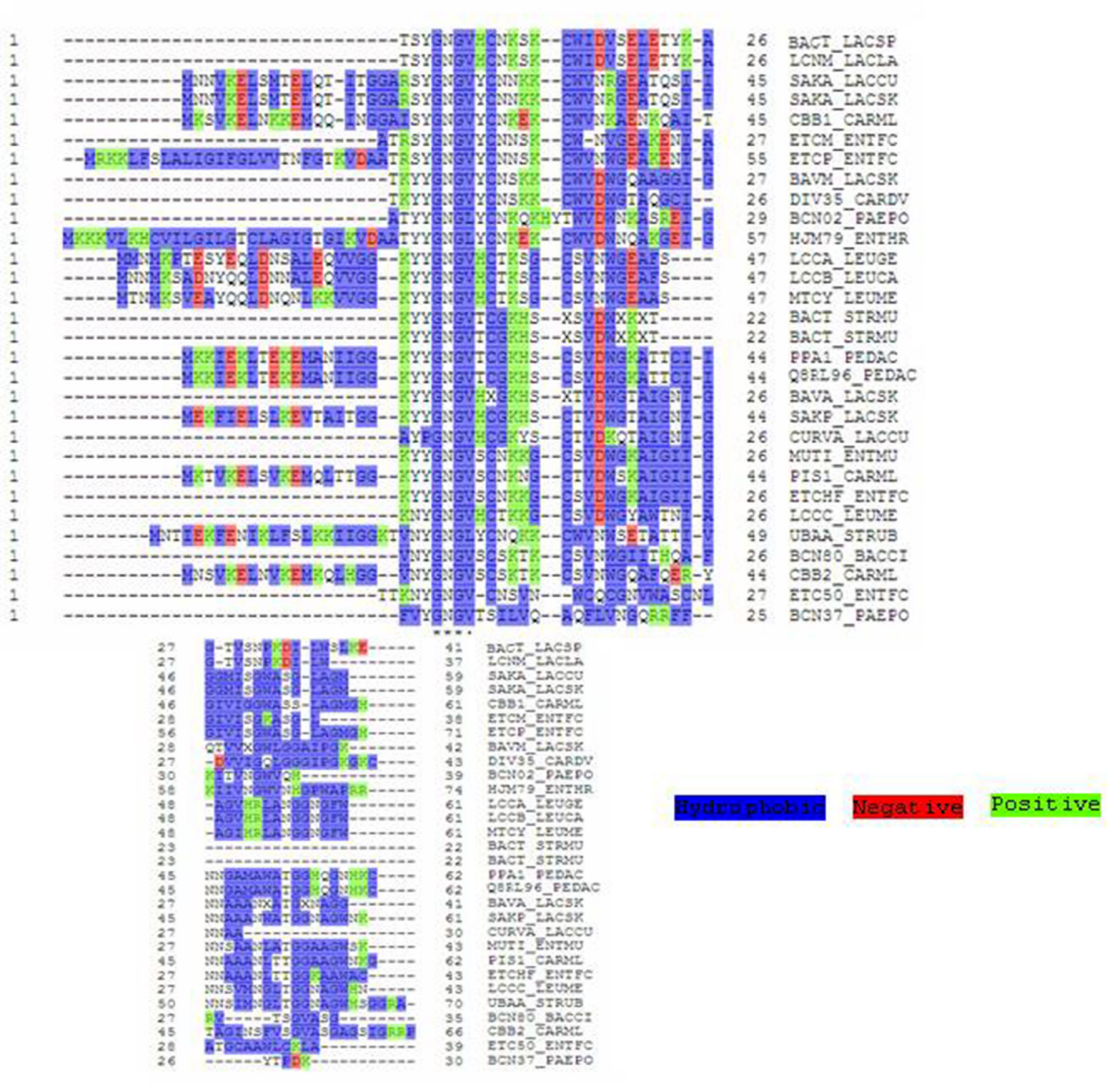
(coloured) Sequence alignment to show the charged amino acids and distribution. Multiple sequence alignment of the YGNGV family showing the conserved regions, and negative, positive, and hydrophobic residues, created with UniProt/SwissProt. The ClustalX program was used to align the amino acid sequences of different ocins. The residues are shaded with the BOXSHADE transferred program. The YGNGV motifs of different ocins are highlighted. The hydrophobic regions are shaded in blue; negatively charged residues in red; and positively charged residues in green. The dots immediately below the alignment indicate the sequences conserved in almost all the ocins. Two dots immediately below the alignment correspond to change of two amino acid residues only. Negatively charged amino acid residues, such as aspartic and glutamic acids, were observed and no other amino acid. Lysines account for more positively charges than other positively charged residues, such as arginine and histidine.

To understand the distribution of amino acids in different ocins, we used the Perl script program (Fig. 3). We found that proline is poorly represented, and alanine, cysteine, aspartic acid, methionine, and tryptophan are sparingly represented. Serine, threonine, asparagine, glutamic acid, and lysine are well represented (Fig. 3). Although the lengths of the ocins differ, the hydrophobic YGNGV motif is highly conserved (Fig. 4). A sequence alignment data also showed that lysine is a conserved upstream region from the YGNGV motif in several ocins. A single cysteine is conserved downstream from the YGNGV motif, which is interrupted by histidine, serine, and tyrosine. Valine, alanine, and isoleucine are conserved downstream from the YGNGV sequence in most ocins. A positively charged ocin is considered a rare phenomenon, but the YGNGV family is unique in this respect. Positive charges are conserved in CBB1, CARML, and HJM79 (Fig. 5). One of the most significant characteristics of the ocins is the presence of cysteine residues, which form disulfide bonds to confer stability.

An unusual phenomenon has been observed in the amino acid distributions of the YGNGV family ocins, as shown in Fig. 3. Proline and phenylalanine residues are poorly represented, as too are aspartic acid and glutamic acid. The percentage representation of different amino acid residues is as follows. Proline, histidine, methionine, arginine, aspartic acid, tyrosine, and tryptophan constitute only 1–5 %. Aspartic acid, glutamic acid, and isoleucine constitute only 5–10 %. The remaining residues, such as alanine, glutamine, lysine, serine, and valine, constitute 10 %. Glycine accounts for 25 % or more, occurring more frequently than proline. Therefore, the YGNGV family ocins are very flexible molecules and this flexibility allows them to change their structure or shapes as required, according to conditions. Their flexibility is attributable to the presence of large numbers of glycine residues. The sequence alignment in Fig. 4 clearly shows the distribution and conservation of the different amino acid residues in the ocins. Overall, the YGNGV motif is highly conserved in all the 29 ocins shown in Figs 3 and 4.

### Comparison with existing databases

ANTIMIC [25, 26] and APD (Antimicrobial Protein Database) are antimicrobial peptide databases and contain proteins from both the prokaryota and eukaryota. The databases contain general information about proteins of all types that express antimicrobial, antifungal, or antiviral activities. However, none of the existing databases, including DBAASP, DRAMP, CAMP, LAMP, BACTIBASE, ANTIMIC, APD, and BAGEL, describe the actions and mechanisms of different types of ocins and their targets. Understanding the natural processes and mechanisms of ocins can help the user to apply them in the food industry. We stress these features because they are essential in the derivatisation of novel second-generation protein-engineered ocins. Therefore, we incorporated the mechanism and action of ocins in the database first page (animations). The well-blogged / frequently accessed databases, such as ANTIMIC, APD and the other databases, do not contain complete information on nisin. We have succeeded in incorporating advanced data of nisin, such as thermostability, thermolability, and susceptibility to essential physiological enzymes. A few biophysical parameters such as free thiols/cysteine residues were also included. BAGEL is a very closely related database that facilitates the prediction of ocins, and can be considered a genome-mining and prediction tool rather than a database, it does not discuss protein sequences, structures, biochemical characterisation, etc.

### Conclusion

The statistical analysis of the whole sequences (sequence alignment through Clustal W) [27] helped us to demarcate the existence of variety of sequences. One among them is YGNGV family. The ocins database has unique features, including information on the YGNGV family, and their functional attributes. The cysteine-rich ocins are also presented in detail. We have taken extreme care to characterise each ocin by constructing histograms that show their actions on different foods originating from various sources, shown as a pyramid. To allow these ocins to be used successfully in the food industry, their resistance and susceptibility to various proteases is included and fully discussed. Their thermostability and thermolability are given because they are of utmost importance to the application of ocins in the food industry. We understand that almost all the ocins are of chromosomal origin, hence, we have not discussed much. UniProtKB/Swiss-Prot, BACTIBASE, ANTIMIC, BAGEL, DBAASP, DRAMP, CAMP, LAMP and APD present large bodies of data, but lack some of the most important features of ocins. One of these is the directions for their use in the food industry; a second is their direct applicability to the food industry, which is directly related to their characterisation. The database is accessed at http://ocins.cftri.com/ocins/.

One of the basic characteristics of the probiotic microbe is the synthesis and secretion of an antimicrobial agent known as bug-buster/polypeptides. All the mechanisms of action of ocins are shown with animations on the main page of the Odb, e.g., from left to right: the mechanism of lantibiotic A, followed of the mechanism of lantibiotic B, and ending with the mechanism of class II ocins (Fig. 2a). A food pyramid is incorporated into the middle of the first page, depicting a series of different food categories. The organisation in the food pyramid is based on the essential requirements for good health and the extent of the consumption of these foodstuffs worldwide. Each layer of the pyramid contains information pertaining to the relevant pathogens, e.g., the first layer of the pyramid contains the bacteria (target organisms) that infect cereals, such as *Achromobacter , Bacillus , Flavobacterium , Micrococcus , Sarcina , and Serratia *. When species of *Bacillus* are selected, the information is provided about the different species of the *Bacillus* and the various types of bacteriocins active against the different species (Fig. 2). To understand the physico-chemical parameters in detail, each ocin can be followed individually. The provider includes the name of the ocin, its mass, pI, net charge, basic and acidic residues, hydrophobic and polar residues, aliphatic residues, Bowman’s index, hydropathy index, aliphatic index, instability index, extinction coefficient, and absorbance at 280 nm. The wheel model and 3D structures are also included, which predict the basic attributes of the protein (Fig. 2). The (predicted) structure of each ocin has been included in the file designated ‘structures available’ on the first page of the database. The contact information is included at the end of the first page. 

## Supplementary Data

Supplementary File 1
