# Supplementary material for: Ocins database: a database of bug-busters from Bifidobacterium, Lactobacillus, and Enterococcus
Source: Access Microbiol. 2019 Jun 13;1(4):e000034. doi: 10.1099/acmi.0.000034 (PMC7470288; doi:10.1099/acmi.0.000034)
Supplement: Supplementary File 1 [file acmi-1-034-s001.pdf]

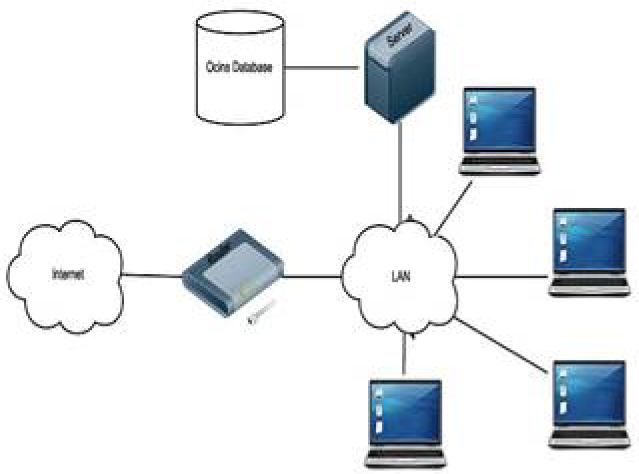

Fig A. Structure of Ocins Database

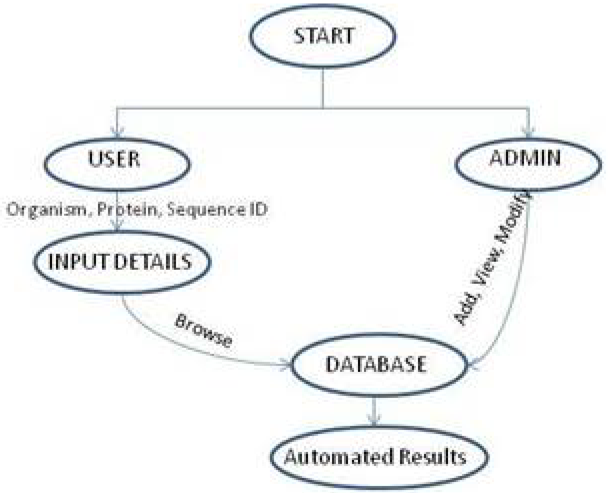

Fig B. The Data-Flow diagram of Ocins Database

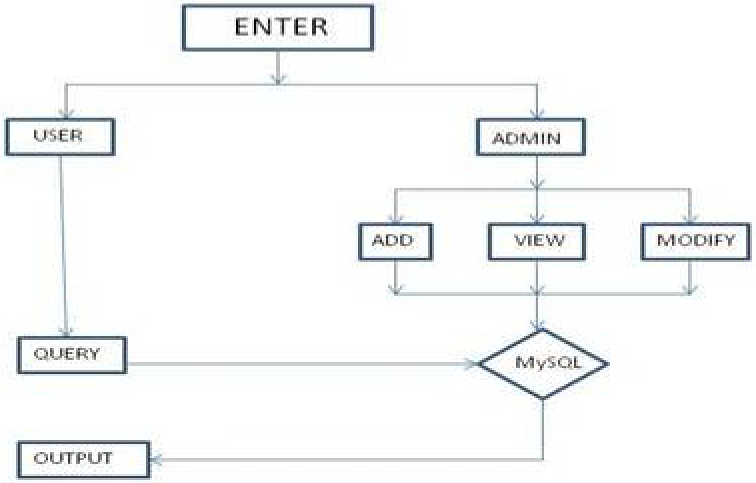

Fig C. Data Schema of Ocins Database

Supplementary material depicting the different stages of construction of Ocins database
